# Diet in Disease

**Published:** 1893-06-24

**Authors:** Ernest Hart

**Affiliations:** Bachelier-ès-Sciences-es-Lettres (rèstreint), formerly Student of the Faculty of Medicine of Paris, and of the London School of Medicine for Women


### Diet in Disease.

By Mrs. Ernest Hart, Bachelier-ès-Sciences-es-Lettres (rèstreint), formerly Student of the Faculty of Medicine of Paris, and of the London School of Medicine for Women.

### XXIV.—URIC ACID AS A CAUSE OF DISEASE, AND ITS PREVENTION BY DIET.

When explaining the part played by albumen in the food supply of the body, it will be remembered that I described how the albuminoids, composed of oxygen, hydrogen, carbon, and nitrogen, yielded up their nitrogen in the process of oxidation in the tissues, which nitrogen was absolutely required for the formation and reconstruction of the tissues and juices of the body without exception: hence the necessity for a certain amount of albuminous food. Now, the final product of oxidation of albumen is urea, which is separated from the blood by the action of the kidneys, and, being a very soluble substance, is dissolved and cast out of the body in the urine. Not all the albumen, however, is oxidised till it becomes urea, a small proportion never reaches this state, and remains only partly oxidised in the blood and tissues in the form of uric acid. Now uric acid is soluble in an alkaline fluid, and much less soluble in an acid fluid. If therefore, by any cause the alkalinity of the blood is decreased, the uric acid present will be driven out of the blood current into the joints, liver, spleen, &c. When subsequently the normal alkalinity of the blood is re-established, the uric acid which has been driven out is washed again into the current, and is present therefore in excess. An excess of uric acid in the blood produces the symptoms of depression of spirits, irritability of temper, headache, and malaise. This whole subject has been very carefully studied by Dr. Alexander Haig, and the results of his inquiry are embodied in a book called "Uric Acid as a Factor in the Causation of Disease." Dr. Haig was led to study this subject by an extremely severe and periodic headache from which he suffered almost every week, and which, from its painful violence and incapacitating character, threatened to cripple or cut short his career. Seeking its cause in order to accomplish its cure, he began to carefully examine the excretions of the body, and he arrived at the following results: That a headache occurred when there was an excess of excretion of uric acid following on a period when there had been a diminished excretion. Uric acid was excreted in excess when the urine was increasingly acid, and presumably when the alkalinity of the blood was high. The excretion of uric acid corresponded with the severe headache; and consequently with the increased alkalinity of the blood and acidity of the urine. Now what are the conditions which alter the alkalinity of the blood and the solubility of uric acid and its consequent excretion by the kidney? Cold decreases alkalinity and drives the uric acid into the joints and tissues; warmth and acid perspiration increase alkalinity, and the uric acid is then washed out of the joints and tissues into the blood; good dinners and generous wines decrease alkalinity, followed by a reaction and falling acidity. Dr. Haig found that by administering an acid to himself he could drive away his headache, by, in fact, driving the uric acid into the joints and producing pain and pricking in these; or he could bring on the headache at will by giving himself a dose of alkali when the uric acid deposited in the joints and tissues was washed out of them into the blood. It was the presence of an excess of uric acid or urates in the blood he therefore argued which caused the headache. He contends that what our forefathers called "phlegm and humors," what the unscientific call "bile," and what the doctors label gout or rheumatism, are all manifestations of the same condition, namely, an excess of uric acid in the system. He believes, however, that this excess is not due to excessive formation of uric, but to decreased excretion. The formation of uric acid is, Dr. Haig states, always as compared to urea as 1 to 33. If, therefore, a person excretes on an average 500 grains of urea a day, he should also excrete about 16 grains of uric acid. If this amount is not excreted it is because it is retained in the body, and will either be found subsequently in the deposits of urate of soda or chalk stones in the joints in gout, or it will be excreted when from some cause the alkalinity of the blood has been raised with the accompanying symptoms of headache, depression, and rheumatic pains. Dr. Haig does not deny absolutely that an excess of uric acid may be formed by deficient oxidisation; but his investigations lead him to believe that urea and uric acid are produced always in the same proportions, and if we want to diminish the one we must diminish the other. They rise and fall together. Now, having arrived at the cause of his periodic headaches, and having formed the opinion that in order to prevent them the formation of uric acid must be diminished, and the high alkalinity of the blood maintained, what was the process of cure adopted? A change of diet. And it is for this reason that Dr. Haig's researches on a rather abstruse subject are particularly interesting to us. His object was to decrease the formation of urea and uric acid, and to keep up the alkalinity of the blood, so that the uric acid formed should be held in solution and steadily excreted daily by the kidneys, instead of being driven into the joints and tissues. Now an animal diet increases acidity, a vegetable diet diminishes it. Dr. Haig succeeded in curing his headaches by reducing the intake of nitrogenous food, and by putting himself on a vegetable diet. In this way the excretion of urea was decreased from an average of 500 grains to 300 grains a-day, and of uric acid from 16 grains to 9 grains. The alkalinity of the blood was maintained, the old stores of urates were washed out of the tissues, and when the exact balance was arrived at, and the uric acid daily produced was daily excreted, the headaches ceased.

Dr. Haig argues that not only the so-called "bilious headache," but also gout, rheumatism, and epilepsy, are caused by excess of uric acid in the blood, and may be controlled by limiting the consumption of animal food and putting the patient on a farinaceous diet. With respect to epilepsy, he considers that the fits are caused by the same condition as produces the periodic bilious headache in others. Dr. Haig conclusively shows that an excess of uric acid in the blood profoundly alters the circulation, and interferes with tissue change and nutrition, which finally results in serious organic disease.

**Mental Depression.**—We are all acquainted with those unfortunate persons who, though they are possessed of all the good things of this world, who, though they have within their reach the pleasures which wealth can give, and the comfort of home and family, yet persist in thinking that life is not worth living, that ruin haunts their steps, and that the affection of friends is not for them. These are the victims of uric acid in the blood, or what is called uric-acidæmia. The condition is graphically described by Dr. Haig: "Self-reliance is absolutely gone, extreme modesty is common, or even habitual, a feather weight will crush one to the dust, and even the greatest good fortune will fail to cheer. If roused from such a condition a considerable amount of irritability and bad temper is sure to be manifested, quite out of proportion to the requirements of the case. . . . Clear the blood of uric acid . . . and the mental condition alters as if by magic; ideas flash through the brain, everything is remembered, nothing is forgotten, exercise of mind and body is a pleasure, the struggle for existence a glory, nothing is too good to happen, the impossible is within reach, and misfortunes slide like water off a duck's back."

In order that this blessed result may be obtained by these sufferers, Dr. Haig lays down the following regime to be followed:—

Animal Food.—Milk 1 to 1½ pints, previously boiled. Eggs, fish, fowl, or game, 1 to 4 oz., varied a little from day to day.

Vegetable Food.—Vegetable-prepared products, vegetables twice a day, fruit three times a day, to any desired extent, according to appetite.

Tea, Coffee, Cocoa in moderation, and as flavourings rather than as strong decoctions.

The daily dietary may be as follows:—

Breakfast.—A large soup-plate half full of porridge eaten with milk; a few mouthfuls of fish or egg prepared in various ways; one or two rounds of bread, or its equivalent in toast, with plenty of butter; a cup of milk, flavoured with tea, coffee, or cocoa, previously boiled. Finish with a small quantity of any fruit that is in season.

Lunch.—Potato and one other vegetable cooked in various ways and eaten with butter, fat, or various sauces; pudding, tart, or stewed fruit; biscuit and butter; a little fruit as at breakfast. For drink a little milk, which in winter is often warm, or water, often taken in summer, with a little fruit syrup, such as Stowers' Lime Juice Cordial.

Afternoon Tea.—Bread and butter and cake of various kinds. A little milk and water flavoured with tea.

Dinner.—Soup made without meat stock; fish, of which only a very small piece is taken; two vegetables with sauces, butter or fat; any ordinary pudding, tart, or stewed fruit, though not as a rule very rich dishes containing many eggs; biscuit and butter; a good supply of various fruits for dessert. For drink, water with syrup, aërated waters, or a little milk, often taken warm in winter; a tumbler of water, aërated water, or in winter hot water at bedtime.

As Dr. Haig states, there is no starvation about this diet; but it has its inconveniences, owing to its running counter to the accepted habits and customs of the country. A mutton chop is always obtainable, while well-cooked vegetables can rarely be got anywhere. But health is worth purchasing at the price of inconvenience and trouble. With an earnestness born of conviction, Dr. Haig asks: "Do we not here in England die younger and in greater number than there is any necessity for? Are we not afflicted with an infinite number of diseases which cause far more pain and misery than is at all necessary? Are we not given to all kinds of debauchery and excess, and have we not huge asylums full of lunatics, and prisons full of criminals?" And he replies to his own queries: "I look upon all these things as serious and widespread diseases in the human race, and as I am not one of those who believe that Nature herself, if she had a free hand, would tend to destroy us, but rather to preserve what is good and eliminate what is evil; and further, cannot believe that the tendency to these evils is part of the ground plan of Nature's work, or that the unalterable bias is to have headache, epilepsy, mental depression, mania, and their results—murder or suicide—alcoholism, morphinism, cocainism, &c., is originally implanted in our nerve centres—I am driven to the conclusion that not a few of these evils are the result of unnatural conditions, and that prominent among these is the unnatural diet, the evil action of which we are now in a position to follow out completely through our knowlege of the powerful effects of urates on the function and nutrition of the whole body." This is a strong denunciation of the meat-eating habits of our race and country.

